# Analysing macroscopic traffic rhythms and city size in affluent cities: insights from a global panel data of 25 cities

**DOI:** 10.1098/rsta.2024.0102

**Published:** 2024-11-13

**Authors:** Martín Saavedra, Alberto P. Muñuzuri, Monica Menendez, Jose Balsa-Barreiro

**Affiliations:** ^1^ Group of NonLinear Physics, Department of Physics, University of Santiago de Compostela, Santiago de Compostela 15782, Spain; ^2^ Galician Center for Mathematical Research and Technology (CITMAga), Santiago de Compostela 15782, Spain; ^3^ Centre for Interacting Urban Netowrks (CITIES), New York University Abu Dhabi, Saadiyat Island, P.O. Box 129188, Abu Dhabi, UAE; ^4^ Division of Engineering, New York University Abu Dhabi, Saadiyat Island, P.O. Box 129188, Abu Dhabi, UAE; ^5^ Department of Geography, University of Santiago de Compostela, Santiago de Compostela 15703, Spain

**Keywords:** commuting patterns, macroscopic traffic flows, mobility behaviour, social physics, traffic rhythms, urban sprawl

## Abstract

The world is undergoing a rapid process of urbanization. Currently, it is estimated that over 55% of the global population resides in urban areas, a figure projected to reach nearly 70% by 2050. This trend is accompanied by a spatial reorganization of human activities on a global scale, bringing about significant changes in mobility patterns and urban traffic management capabilities. Consequently, it is imperative to evaluate, on a broad scale, how city size influences traffic capacity. This study aims to analyse on-road traffic patterns using a diverse dataset comprising cities of varying population sizes, geographical extents and global locations. Specifically, we conduct an analysis encompassing 25 cities primarily situated in several European countries (France, Italy, Germany, Spain, Switzerland, and the United Kingdom), as well as in North America (Canada) and East Asia (Japan and Taiwan). Our findings shed light on how physical aspects related to urban form influence mobility patterns, offering insights for the implementation of more effective and sustainable traffic management policies.

This article is part of the theme issue ‘Co-creating the future: participatory cities and digital governance’.

## 1. Introduction

In the current landscape marked by escalating mobility rates attributed to economies of scale [1], traffic congestion poses a pervasive global challenge. In the United States, road mobility has increased by 60% in the last three decades, escalating from 3.9 in 1990 to 6.2 million passenger-kilometres in 2021 [2]. This trend aligns with ongoing urban expansion, where increased wealth correlates with higher motorization rates. As a result, major global economies are experiencing heightened levels of congestion [3,4].

Traffic congestion has a widespread impact, extending beyond specific areas and causing a ripple effect that affects entire urban networks and other drivers [5,6]. Traffic jams lengthen commute times by amplifying energy consumption and worsening environmental conditions, such as air pollution, noise and temperature levels [7]. The US Environmental Protection Agency [8] estimates that on-road vehicles account for 82.7% of all the emissions from the transportation sector, with 40% of these emissions concentrated in cities [9]. Traffic jams not only pose significant health hazards due to emissions [10], but they also increase the risk of accidents and fatalities [11], while obstructing accessibility to essential emergency services [12].

Estimating the total costs of traffic congestion remains challenging, despite the pioneering efforts of scholars like Pigou [13]. A common approach is to calculate the extra travel time beyond ideal free-flow conditions and express it in monetary terms. Kim’s analysis [14] shows that the average US commuter spends an additional 8.1% of their daily commute time, or 51 h per year, costing about $869 [15]. In short, annual costs are estimated at $179 billion in the United States [16] and €110 billion in Europe [17]. These costs vary significantly across regions due to the uneven distribution of traffic congestion. For instance, these costs represent 1% of the total European gross domestic product (GDP) [17]. More extreme values are observed in large metropolitan areas such as Beijing, China, where congestion costs reached 4.2% of GDP just a decade ago [18].

Private cars pose challenges because of their high resource demands and environmental impact, especially in densely populated areas with limited public space. Managing traffic congestion is a multifaceted challenge that resists seemingly intuitive solutions, such as increasing transit capacity by constructing more roads [19]. The *Pigou–Knight–Downs* and *Downs–Thomson* paradoxes illustrate the inefficacy of expanding road capacity to alleviate congestion, as this can lead to the counterintuitive outcome of stimulating more demand [20]. From 1993 to 2017, the United States spent $500 billion on expanding highways in the 100 largest urban areas. Despite increasing highway capacity by 42% to support a 32% larger population, traffic congestion (total annual hours of delay) surged by 144% [21]. Instead of expanding road capacity, cities dealing with traffic jams often adopt control strategies based on congestion pricing systems to restrict access to city centres while encouraging public transportation [22]. However, criticisms persist. Some studies suggest that enhancing public transport might not necessarily alleviate traffic congestion [23], whereas implementing congestion taxes for private cars may disproportionately affect vulnerable social communities [24].

Traffic congestion predominantly affects urban areas. A recent report on 278 Asian cities found that city size jointly explained 64% of congestion variance. However, urban dwellers often rely less on cars in favour of public transport and active mobility modes [25]. This shift is driven by a combination of diverse factors including fuel prices, transit services, technological advancements, socio-economic trends and policy regulations, whose impacts vary by city size [26]. These factors influence traffic dynamics, potentially decoupling traffic congestion from city size, as evidenced by the concerning surge of traffic jams in medium-sized cities [27], where people rely more on cars due to convenience and the lack of alternative mobility options [28]. Therefore, a thorough analysis of how city size influences traffic patterns is essential yet challenging.

For this purpose, we analyse on-road traffic rhythms considering city size. We collect population data in city cores and metropolitan regions to estimate commuting population. Traffic dynamics are assessed using macroscopic daily flows extracted from a wide network of detectors placed in various parts of each city. We evaluate these dynamics across a diverse panel of cities, distributed across different regions, with varied populations and sizes.

The rest of this article is organized as follows: §2 conducts a literature review that explores the relationship between built environment and mobility behaviour. Section 3 introduces the dataset and study areas, while §4 outlines the methodology employed. Section 5 presents the findings, and §6 critically discusses and interprets them. Finally, we include a list of limitations to provide a more rigorous interpretation of the results.

## 2. Built environment and mobility behaviour

Large accessibility to private automobiles and continuous investments in road capacity have driven urban sprawl, allowing people to live farther from city cores [29]. Urban sprawl has lengthened daily commutes and increased mobility rates, resulting in higher levels of traffic congestion [21]. Louf & Barthelemy [30] found that congestion sensitivity is affected by factors such as distance driven, road network length and *$CO_{2}$* emissions. They observed that as larger urban areas developed, congestion points shifted away from city centres. Cities with intensified land use and polycentric structures tend to experience more traffic jams [31]. Consequently, traffic congestion often promotes urban sprawl and the development of less densely populated areas, creating a feedback loop between traffic dynamics and urban form [32]. While most studies assert that urban sprawl increases commuting distances, in addition to traffic congestion, parking issues and road fatalities [33,34], other studies argue that development sprawl may have the opposite effect, potentially reducing commuting times [35]. This suggests that the relationship between urban sprawl and traffic externalities remains ambiguous.

On the other hand, compact and densely populated cities rely less on private cars [28], promoting public transport and active mobility [25]. In compact cities, public transportation is more feasible and accessible due to shorter distances between origins and destinations and a demand that is heavily concentrated [36]. In the last years, urban mobility in city cores is being restructured to further reduce the environmental impact and resource consumption of private cars. Efforts are focused on enhancing ride-sharing, mass transit and more efficient transport systems to rebalance the modal share [26]. These changes are driven by initiatives that elevate operational costs for driving such as congestion pricing, limitation of parking slots and stricter emissions regulations to discourage car use [37] and facilitated by technological advancements like ride-sharing apps and electric vehicles, which have made commuting more flexible and eco-friendly [38]. Besides that, recent social changes, such as the widespread adoption of telecommuting and shifts in the labour market post-COVID-19, have contributed to urban sprawl by reducing daily commutes and altering peak traffic flows [39].

Changes in urban mobility are transforming public spaces. Most western cities are reducing space for cars and developing green infrastructure for promoting active mobility, such as bike lanes and pedestrian zones, while many others are fostering compact mixed-use developments to reduce travel distances and enhance urban mobility efficiency [40]. These initiatives require major mobility plan changes, promoting multi-modal systems that combine public transport with popular micromobility options, as seen in Europe, where 270 million people travelled 535 million kilometres on electric scooters and bicycles in 2023 [41].

The relationship between traffic dynamics and city form must be understood reciprocally. Transportation systems have contributed to modifying city layouts. For example, the dense configuration of narrow streets that dominated medieval European cities, optimized for horse traffic and pedestrians, was replaced by expansive boulevards designed for automobile traffic in the early to mid-twentieth century [42,43]. Nowadays, topology of urban networks determines various aspects of social behaviour, including mobility patterns [44]. The spatial configuration of urban networks and city layouts allows for evaluating parameters such as connectivity, axial integration and accessibility using spatial syntax methods [45]. These parameters can predict areas that are vulnerable to traffic congestion. Thus, Choi & Ewing [46] found that neighbourhoods with denser and more connected road networks experience less congestion. Christodoulou & Christidis [47] observed that congestion patterns in Sevilla, Spain, mirror population distribution and transport demand, with higher car commuting rates in low-density suburban areas. In summary, aspects related to city size and urban form play a crucial role in understanding commuting patterns in terms of travel distances, car ownership and modal choices [26,48,49], which allows for predicting traffic dynamics and vulnerability to congestion.

## 3. Data and study area

Comparing mobility across cities and countries is challenging due to unreliable information and a lack of standardized methodologies. These challenges include defining study areas and the scarcity of comprehensive datasets or reliable global reports [26]. In this article, we compile traffic data based on lane occupancy from 25 cities across the globe. This information is extracted from the largest publicly available multi-city traffic dataset [50,51]. Despite the considerable differences in size and population, all the cities in this dataset are located in affluent nations. In short, this list includes 22 European cities: Graz in Austria; Bordeaux, Marseille and Toulouse in France; Augsburg, Bremen, Darmstadt, Constance, Essen, Hamburg, Kassel, Speyer, Stuttgart and Wolfsburg in Germany; Cagliari in Italy; Madrid and Santander in Spain; Basel, Bern, Lucerne and Zurich in Switzerland; and London in the United Kingdom. Additionally, three cities outside Europe are also included in the list: Toronto in Canada, Tokyo in Japan and Taipei in Taiwan.

In this section, we introduce the different datasets categorizing them into two subsections: §3a provides information on how we delimit these cities and extract their population data, while §3b focuses on data related to on-road traffic. Further details for each subsection are outlined below.

### (a) City delimitation and population data

Our initial step involves delineating our study areas. To achieve this, we consider the boundaries separating cities from metropolitan areas. In our study, we demarcate regions using the *Global Human Settlement Layer* (GHSL), which provides global population data in a raster format [52]. The outcomes for the year 2020 are visually depicted in the electronic supplementary material, figure S1, while the compiled numerical data are summarized in the electronic supplementary material, table S1. Based on GHSL’s spatial raster dataset, urban regions are separated into two groups: Urban Centres (UCs) and Functional Urban Areas (FUAs). A UC constitutes a cluster of cells hosting at least 50 000 inhabitants and a density of at least 1500 inhabitants per $km^{2}$, potentially encompassing cells with at least a 50% built-up area. This initial cluster is refined through internal gap filling and edge smoothing techniques. On the other hand, a FUA includes the urban centre and its commuting zone, identified as regions where at least 15% of the population commutes daily to the urban centre [53]. Throughout this study, we will interchangeably refer to FUAs or *metropolitan areas*.

In the cases of Essen and Speyer, official delineations were not available. To address this concern, we establish an arbitrary delimitation method. First, we estimated the *Urban Core* as the area occupied by the administrative region of its municipality. To delineate the metropolitan region (FUA), we determine the position of a centroid, around which we estimate an area of influence equal to the maximum distance of the aforementioned administrative boundaries. For Speyer, this distance was 5.9 km, whereas for Essen, it was 10.4 km. With these values, we estimate population values sourced from national institutions. In Constance, official data initially reported less population in the FUA (85 218 inhabitants) than in the UC (87 030). Upon re-evaluation of the UCs and FUAs, discrepancies were observed. Once these were adjusted, the FUA increased from 182.7 to 207.3 $km^{2}$, and its population estimate was increased to 106 220 inhabitants.

Population data shown in the electronic supplementary material, table S1, highlight five large metropolises, namely, Tokyo, London, Taipei, Toronto and Madrid, each with over 5 million residents. Hamburg and Stuttgart have populations surpassing 1 million, while Zurich, Marseille, Toulouse and Bordeaux reach this mark within their FUAs. Metropolitan areas of Basel, Bremen and Augsburg range from half a million to a million inhabitants, with Bremen standing out at over 900 000. Mid-sized cities have populations between 200 000 and 300 000, and smaller cities, notably in Switzerland and Germany, have fewer than 200 000 residents. German and Swiss-German cities are more widespread showing higher ratios between population FUA/UC compared with large metropolises and port cities.

Tokyo (8025 $km^{2}$), London (4796), and Toronto (3843) are the most widespread cities in terms of area, mirroring this pattern to their metropolitan regions, which exceed 15 000 $km^{2}$. Taipei (2833) stands as an exception, with a smaller FUA compared with many central European cities. Central European cities exhibit significantly higher ratios, around 10-fold, comparing FUA size with UC, while southern European cities present ratios around 5–6. Large metropolises have the lowest ratios, such as Toronto (4), London (3.5), Tokyo (2.2), and Taipei (2.4).

Large metropolises like Taipei (7613 inhabitants per $km^{2}$) and Tokyo (4292) are the most densely populated. The rest of the large metropolises have relatively high population densities, which in the case of the metropolitan area of Madrid is comparable with those of Tokyo. Mid-sized southern European port cities like Cagliari, Marseille, Santander and Bordeaux present relatively large population densities, exceeding 1500 inhabitants per $km^{2}$. Meanwhile, mid-sized Swiss and German cities generally have population densities ranging between 1000 and 1500 inhabitants per $km^{2}$, with lower densities in their metropolitan areas, ranging from 200 to 400 inhabitants per $km^{2}$.

In summary, our dataset showcases diverse urban characteristics across regions. North American cities, like Toronto, exhibit urban sprawl driven by widespread car ownership and infrastructural expansion, resulting in extensive suburban growth. European cities emphasize compact urban designs with sustainable infrastructure and walkability, while preserving open spaces and promoting community life. East Asian cities like Tokyo and Taipei feature densely populated urban centres with substantial growth in nearby satellite cities, driven by factors such as affordable suburban land and extensive use of public transportation systems. Urban redevelopment efforts have transformed these cities, fostering modern districts and mixed-use zoning. Large population densities lead East Asian cities to prioritize upward growth over outward expansion, setting them apart from Western cities.

### (b) On-road traffic data

On-road traffic data were compiled from the UTD19 database [50,51], the largest publicly available multi-city traffic dataset. This dataset contains on-road traffic information collected from 2017 to 2019 through a network of 23 767 stationary loop detectors located in 41 cities [54]. Each loop detector recorded the number of vehicles (flow-occupancy measurement pairs) on each roadway at intervals of less than 5 min. Although the spatial distribution of loop detectors varies among cities, many of them were located on road intersections, facilitating traffic flow evaluation on major thoroughfares. As loop detectors can be prone to malfunctions [55], the original dataset underwent refinement and filtering by Bramich, Menendez and Ambühl [56]. The refined dataset consists of data collected from 10 150 loop detectors spanning 25 cities, comprising more than 147 million records. In the electronic supplementary material, table S2 presents a consolidated summary of the UTD19 refined database.

**Figure 1.**
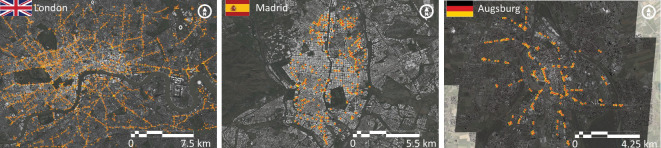
Spatial distribution of loop detectors across London, Madrid and Augsburg. The map of loop detectors shows their spatial distribution across urban networks. London has the largest number of detectors with widespread coverage throughout the city. By contrast, Madrid’s detectors are concentrated in the urban core, while Augsburg’s detectors are mainly located on the access roads to the city centre.

The spatial distribution and configuration of loop detectors’ networks across cities determine to some extent the individual measurements. In fact, due to traffic regulations in most cities, the placement of detectors on urban roads is almost exclusively associated with private and small commercial vehicles. Besides that, loop detectors tend to yield a large proportion of measurements acquired during non-stationary traffic states typically associated with traffic jams due to flow interruptions, caused—for instance—by road intersections. This explains the significant presence of noisy measurements. In the electronic supplementary material, figure S2 illustrates the spatial distribution of loop detectors for all cities, while figure 1 displays a zoomed-in view focused on the most central regions of three example cities.

Two of the largest metropolises, London and Tokyo, boast the widest coverage of loop detectors, with 4096 and 1295 detectors, respectively, ensuring substantial monitoring across their complete urban networks. Madrid maintains a moderate coverage with 325 detectors, primarily concentrated in the city core. Taipei and Toronto account for much fewer detectors (84 and 70, respectively) but extensive coverage in their central areas. Excluding London and Tokyo, Zurich boasts the highest coverage (761), while Bern exhibits the highest density (2.39 $detectors/km^{2}$). At the other end of the spectrum, we find eight cities with fewer than 100 detectors, with Essen having the lowest count (29). Despite varying detector counts, most cities feature spatially spread distributions within their central areas, while Toronto, Basel, Lucerne, Constance and Wolfsburg exhibit a more concentrated distribution in some particular city regions and/or streets.

## 4. Methodology

The methodology for this study is introduced in three subsections, as follows: §4a explains the algorithms to extract traffic flow patterns; §4b details how the correlation coefficients between traffic flow patterns and time lags; and §4c presents the methodology to obtain the relationship between the width of the input tails and commuting population in each city. A more detailed explanation can be found in the electronic supplementary material, section S3.

### (a) Data processing

Loop detectors, embedded in road surfaces, estimate traffic flows by detecting changes in inductance caused by vehicle ferromagnetic materials [56]. Each city has a number of loop detectors ($N_{LD}$) distributed throughout its urban network. These detectors collect traffic flow data over specific time intervals (Δt) for a number of days ($N_{days}$), so each detector provides a time series of traffic flows per day. Each city varies in the number of detectors, the days measured by these detectors and the measurement time intervals, as shown in the electronic supplementary material, table S2.

The study of macroscopic traffic dynamics across cities requires merging the information provided by all loop detectors through a two-step process for each individual city. In the first step, we average the traffic flow data obtained by each loop detector for each of the days. To perform this averaging, we use the arithmetic mean, considering that the data coming from each detector are samples from the same distribution. This allows us to obtain a less noisy time series of data for each loop detector. In this procedure, we only consider weekdays, as traffic flow patterns can vary significantly on weekends in some cities.

In the second step, we standardize the time series (i.e. normalize them to a mean of 0 and a standard deviation of 1) and use the *K-shapes clustering algorithm* [57] to group the time series from each loop detector based on their shape, rather than their magnitude or lag. Once the different patterns are classified, we compute the Euclidean barycentre of each cluster (each set of time series grouped by the algorithm), which represents a good estimator of the average time series.

With this, we obtain $N_{cluster}$ traffic patterns for each city. The patterns found can always be divided into two types: (i) *inflow patterns*, those with a peak flow in the hours close to the early hours of the day and (ii) *outflow patterns*, with a peak in the late hours of the day. In this article, we focus on the inflow patterns, as they are more suitable for the analysis of the entry tails. A more detailed overview of the methods employed and the patterns observed can be found in the electronic supplementary material, subsections S3.1 and S4.1.

### (b) Correlations of traffic flow patterns between cities

After obtaining the main time series of traffic flow patterns, we are interested in examining the similarity between patterns of different cities. To do this, we use the cross-correlation function and the correlation coefficient.

**Figure 2.**
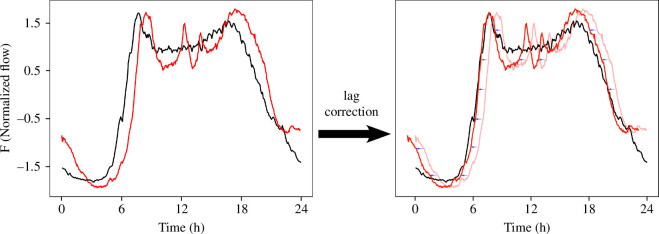
Figures show the process of superimposing the traffic patterns of different cities correcting the time lag computed through the correlation function.

The *correlation function* allows us to find the lag between two time series that maximizes their similarity. This lag is what we call in our context the time lag between traffic flow patterns (see figure 2). Once the time series of two cities are superimposed (lag corrected), we can compute the correlation coefficient between them in order to quantify their relationship. A detailed version of these calculations can be found in the electronic supplementary material, subsection S3.2.

Values of the correlation coefficient and time lag for each city are stored in matrices, which will be symmetric and antisymmetric, respectively. Their diagonal elements will be equal to 1 and 0, since the correlation of a city with itself is maximum and the time lag is 0. We then use these matrices as similarity matrices to conduct a hierarchical clustering. This allows us to group cities with similar traffic flow patterns and time lags to better visualize the similarities between them. We use Euclidean distance as a metric to perform the clustering.

### (c) Temporary size of the entry tails

Our results reveal a common pattern across cities: during the early morning hours, traffic flow starts at minimum levels and steadily rises to peak levels. This trend corresponds to the city’s traffic ‘awakening’. We refer to this early upsurge in flow as the *entry tail*.

The width of the entry tail can be understood as a measure of the total time it takes for each city to absorb all the incoming traffic flows. This entry tail has different widths depending on the city observed. We are interested in studying how the commuting population may affect this amplitude, so, to show this, we calculate the width of each entry tail (see the electronic supplementary material, subsection S3.3) and plot them against the commuting population in each city. We then compute *Spearman’s* rho *correlation coefficient* between these two variables.

To obtain the confidence intervals, we use the bootstrapping method. We resample the data 100 000 times and compute the correlation coefficient for each resample. The confidence interval is then obtained by taking the 2.5 and 97.5 percentiles of the distribution of the correlation coefficients computed.

## 5. Results

This section is divided into three subsections, each presenting the results from the methodology previously described: §5a presents the traffic flow patterns found in each city; §5b shows the comparison between traffic flow patterns through the correlation coefficient for each city pair and the time lag obtained; and finally, §5c presents the influence of commuting population on the size of the entry tails.

### (a) Traffic flow diagrams

**Figure 3.**
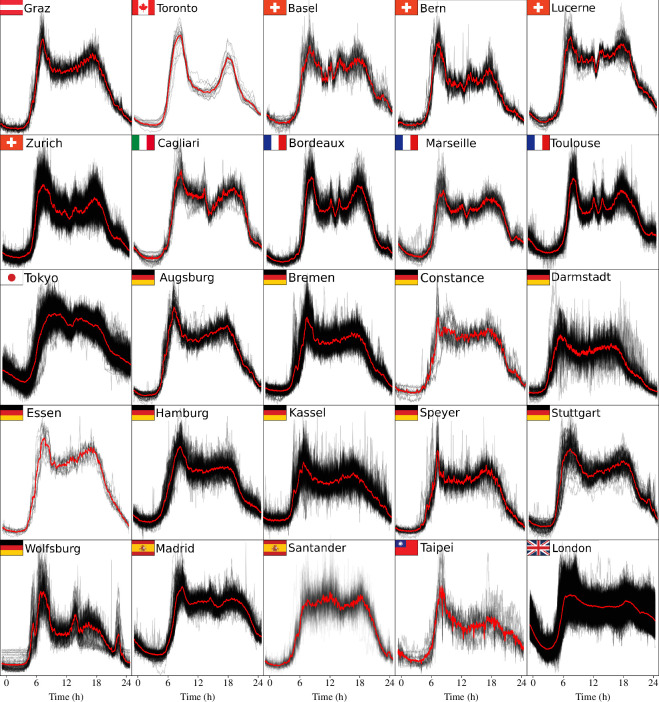
Traffic flow patterns across cities. In the background, black lines represent all time series from each loop detector. The solid red line represents the Euclidean barycentre. This figure shows only the inflow patterns (and the time series belonging to the inflow cluster). The complete figure, with all the flow patterns per city, can be found in the electronic supplementary material, subsection S4.1.

Figure 3 depicts a comprehensive overview of the daily traffic patterns. All the traffic flow patterns exhibit remarkably similar traces, showing alignment between the end of one day and the beginning of the next. This is consistent with the fact that only weekdays were taken into account, so the traffic flow follows the same distribution every weekday.

Going into more detail, certain special motifs can be observed between cities belonging to the same country. The French cities exhibit a similar pattern during the middle of the day, with two small flow peaks and a marked decrease in traffic flows. The same applies to German cities, which display very similar patterns. The exception is Wolfsburg, which shows a much larger decrease in flow after the morning peak and a pronounced peak in the late hours. Similarly, the pattern observed in Graz resembles that of the German cities. Swiss cities, on the other hand, have their own distinct patterns, with varying degrees of similarity to both German and French cities. Finally, Spanish cities show three flow peaks: one in the morning, another at midday and the last in the evening. In Santander, the morning peak is the least pronounced.

### (b) Correlation between flow patterns and time lag

**Figure 4.**
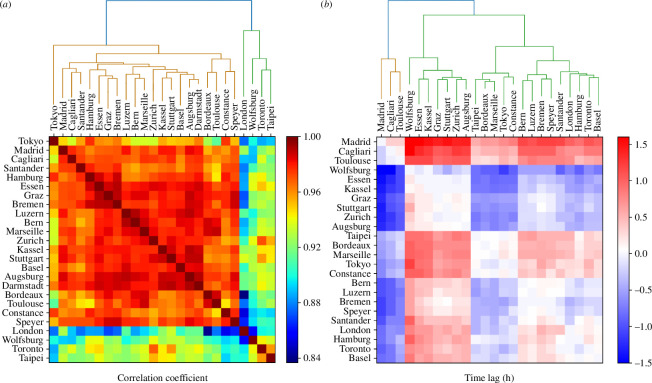
Heat maps displaying the correlation coefficient obtained between (*a*) the traffic inflow patterns of each city and (*b*) the time lag determined to obtain this correlation. The order of the cities in the visualization is determined by hierarchical clustering based on correlation (*a*) or time lag (*b*). The dendograms obtained from the hierarchical clustering are shown above each panel. The city of Darmstadt is not included in figure (*b*) due to its excessively anomalous lag value compared with other cities.

According to the methodology introduced in §4b, we can mathematically corroborate the similarities between the flow patterns of the cities studied by means of the correlation coefficient. The results are represented in the heat maps shown in figure 4.

The results reveal high correlation values in all the cases, with a minimum value of 0.83. This indicates that all the cities exhibit very high levels of similarity in their macroscopic traffic flows, regardless of their differences in city size, population, geographical location and urban form.

We search for similarities between groups of cities using hierarchical clustering. The results shown in figure 4*a* reveal an initial division into two blocks: the group consisting of London, Wolfsburg, Taipei and Toronto; and the rest of the cities. These four cities in the first block show the lowest correlation values compared with the rest. Even among themselves, the correlation values are not very high, with Toronto and Taipei being the most similar in terms of traffic flow patterns. London stands out as the city most different from all the others.

In the second group of cities, we observe that cities within the same country tend to show greater similarities (Bordeaux-Toulouse-Marseille, Madrid-Santander, Lucerne-Bern-Zurich-Basel, ...). However, internal differences are observed within countries, as seen with the cities of Constance, Speyer and Wolfsburg in Germany. Within this block, Tokyo shows the largest differences from the rest.

Figure 4*b* presents the value of the time lags between traffic flow patterns. From this figure, we can distinguish two major groups (unlike the previous ones) and up to five minor groups with slight differences between them. We note that most of the cities of the same country belong to the same main cluster, but there is some deviation, as for example in the case of Basel with the rest of the Swiss cities; Constance, Speyer and Hamburg with the rest of the German cities; and Madrid with Santander.

The city of Darmstadt shows very outlier behaviour, exhibiting a much more prolonged time lag starting approximately 3 h earlier than the other cities (see the electronic supplementary material, figure S2). Thus, we consider this city an outlier and exclude it from the previous figure. For a more complete perspective, a revised version of the figure with all cities, including Darmstadt, is shown in the electronic supplementary material, section S4.2.

### (c) Correlation between commuting population and entry tails

**Figure 5.**
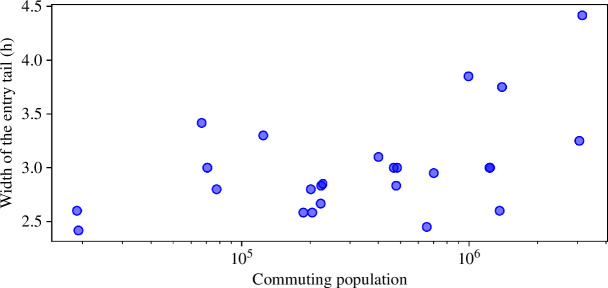
Correlation between the width of the entry tails and the commuting population across cities. The Spearman correlation coefficient is ρ=0.46 with confidence intervals (95%) of [0.01–0.78], *p*-value = 0.018.

At this point, we evaluate how the commuting population could impact traffic flows. For this, we correlate the width of the entry tails, i.e. time, versus the estimated commuting population in each city. This population is identified based on the commuting zone, which extends from the FUAs. This zone encapsulates the economic and functional boundaries of the city, shaped by daily movements of people and closely linked to the city’s job market [58]. Thus, we calculate the commuting population as the population residing within the FUA, excluding those living in the city centre. The results are shown in figure 5.

The results from the correlation yields a value of Spearman’s ρ=0.46 with confidence intervals (95%) of [0.01–0.78], p-value = 0.018. Hence, any ρ-value within our confidence interval confirms the statistical significance of a positive correlation between the width of the entry tails and the commuting population. While the outcomes suggest a logical pattern, the scaling process is not entirely linear. The results show that there is a significant positive correlation between the commuting population and the size of queues entering cities. While this may seem obvious, the distribution of the data may show an interesting insight.

We can observe that the size of the entrance queues seems to grow to a greater extent in those cities with more than 1 million commuting population, while below this population the entrance queues do not show such a clear increasing trend. If this behaviour is indeed real, it could indicate the existence of a critical commuting population value above which cities are not able to efficiently handle the incoming traffic flow. This translates into an increase in the size of the entry tails (around $10^{6}$ in our results), most likely due to an increase in traffic congestion at city entrances. Below this critical value, we would find cities that are able to handle this commuting population, resulting in a ‘constant’ (low slope) value of the entry tail width. Even so, greater precision and a larger number of cities would be necessary to confirm this hypothesis.

## 6. Discussion of results and conclusions

In this article, we evaluate how on-road traffic rhythms relate to general parameters such as the city’s size and population. These parameters help determine aspects of the city’s model, as well as its structure and urban form. We plot traffic flows from 25 cities over a regular working day using data from loop detectors. These plots show how each city’s network handles traffic based on its capacity. Analysing on-road traffic data, this study provides insights into the efficiency of urban networks in managing traffic flows. This is particularly relevant given the significant changes in urban mobility and efforts to reduce private car usage.

This study aligns with recent studies evaluating how urban forms influence traffic dynamics [25,49]. However, our study introduces two novel aspects: (i) we compare a diverse and large group of cities across three continents with varied urban structures reflecting geographical heterogeneities on mobility patterns and (ii) we analyse quantitative and extensive traffic flow data with high spatiotemporal resolution for entire cities.

The diversity and heterogeneity of cities included in this study provide valuable insights. We include small and mid-sized European cities, along with large global cities such as Madrid, London, Toronto, Tokyo and Taipei. The first group of small and mid-sized cities is very heterogeneous due to cultural differences between countries. Despite being understudied, this kind of city accommodates the majority of Europe's population [59] and their population size makes them more conducive to a prominent role for private cars. By contrast, the second group of larger cities, which are much more studied, are of great interest due to their dynamic efforts to improve mobility and address the high costs and risks of traffic congestion. These cities are all located in prosperous countries with regulated urban growth, typically featuring organic development and orderly spatial layouts [60]. As a result, they generally exhibit high accessibility and connectivity across their urban networks [45]. The exception is the two East Asian cities, which have experienced a rapid and partially chaotic urban growth in certain areas due to accelerated urbanization processes and high levels of agglomeration over the past few decades [61].

Urban mobility depends on city size and population density, which strongly influence mobility choices and the feasibility of different modes of transport [26]. For example, long commuting distances in large cities significantly complicate finding alternatives to car commuting, as a more widespread or insufficiently dense population hampers the efficiency of public transport in terms of service [62]. Conversely, small or mid-sized cities often rely more on private vehicles, where public transport does not offer always a competitive option to private cars [26].

Regarding the results presented, we can discuss three main points. First, the analysis of results varies depending on the scale. At the largest scale, all cities exhibit very similar patterns with two marked peaks, one in the morning and another in the afternoon/evening, coinciding with the influx and exodus of workers from their workplaces. This similarity illustrates the largely predictable nature of human mobility [63], as evidenced by repetitive macroscopic traffic patterns observed across multiple cities [64]. Upon closer inspection, significant differences between cities become apparent based on how we aggregate the data. Cities with similar cultural affinities, population sizes, or even geographical location, among other factors, tend to resemble each other. For example, Germanic cities (those located in Germany, Austria and German-speaking cantons in Switzerland) reveal substantially similar behaviours, marked by distinctive peak patterns, including robust daytime peaks occurring at similar times, and a noteworthy peak at evening. The same applies to the largest populated cities, which show very similar trends based on greater vulnerability to congestion, favouring mass transit systems that make them more alike to each other than to cities with similar cultural backgrounds. Significant differences are also observable by geographical location, which determines urban forms, cultures and socio-economic models. For instance, southern Mediterranean cities (Italian and Spanish cities) exhibit their own similarities, and a similar pattern can be seen in coastal cities, where geographic constraints dictate and limit the direction of the city's physical expansion.

A detailed characterization of the main clusters shown in the results reveals interesting patterns in modal share [65], highlighting differences in private car reliance that may influence the traffic capacity of urban networks for each city. Germanic cities are characterized by significant public transport usage regardless of their population size. Within this group, Swiss cities predominantly use alternatives to private vehicles (over 75%), with private vehicle usage just over 20%. Similar patterns with a slightly higher reliance on private cars are observed in Germany and Austria. The major difference between Swiss and German cities lies in the weight of public transportation, which is more substantial in Switzerland, with percentages above 25% in all cities. In Germany, these percentages are below 20% in all cities, except for Hamburg, Kassel and Speyer. Southern European cities exhibit similar patterns, with more reliance on cars and less on public transport. Compared with Germanic cities, French cities show a much higher reliance on private cars, ranging from 54% in Marseille to 75% in Toulouse, with public transport usage accounting for a maximum of 11% in Marseille. A similar pattern is observed in mid-sized southern European cities like Santander and Cagliari, with public transport usage around 15%, though car reliance is significantly lower in Santander (56 versus 66%).

Large metropolises share characteristics such as the minimal use of private cars in favour of extensive public transport systems, ranging from 24% in Toronto to 51% in Tokyo. This relatively wide range must be understood within the specific context of each city and region [66]. Toronto is a very widespread and car-centric city, with almost 70% of trips made by car and minimal active mobility (6%) due to long distances. The two European metropolises show significant differences. London has a very high reliance on public transport (45%), with private cars accounting for only 27% of trips, slightly lower than active mobility at 28%. Madrid’s modal share is similar to other southern European cities, but with slightly lower private car usage (40%) due to a higher share of public transport (25%). Lastly, the high densities of East Asian cities result in the dominance of public transport (43% in Taipei and 50% in Tokyo), thanks to having two of the busiest metro systems in the world. The main difference between these two cities is the substantial disparity in private vehicle usage, with Tokyo having a minimal rate of 12%, compared with 40% in Taipei, where motorcycles are a common mode of commuting [67].

Second, traffic capacity is defined by city size, but also constrained by urban form, which plays a significant role in the propagation of traffic congestion throughout the urban network [68,69]. Hence, the shape of the initial curve and length of entry tail represent the traffic absorption capacity of the urban network, while the slope provides insights into congestion levels. We have observed how entrance queues tend to grow more in cities with over 1 million commuters, while cities with fewer commuters do not show this clear increasing trend. The patterns obtained can be cross-validated with external data sources on commuting times and traffic congestion. According to the OECD [70], the average commuting time (considering all transport modes) varies significantly: North American cities, which are more car-centric and sprawled, have times around 25 min; East Asian cities, which are denser and rely heavily on mass transit systems, exceed 50 min. European cities, more diverse in urban structures and modal share, average about 38 min. Central European countries like Germany have longer commuting times, while southern countries have shorter times. Results show that public transport commutes are 30–40% longer than car trips [70], highlighting the trade-off between resource efficiency and longer travel times. Besides that, on-road traffic congestion data from INRIX [15] and TomTom [71] indicate that large metropolises and French cities are the most congested. This comparison can serve as cross-validation for the cities included in this study, while also providing insights for other cities with similar traffic patterns [72].

Third, the results presented provide valuable insights into various macroeconomic aspects related to urban dynamics, economic models and labour productivity. Financial, technological or administrative cities exhibit distinct mobility patterns compared with industrial cities. For instance, port cities display a more extended curve, while cities with a substantial administrative presence exhibit more concentrated and shorter entry tails. Darmstadt stands out with significant night-time mobility, which could be due to the impact of international research centres and high-tech companies located there. Analysing patterns, the temporal gap between morning and afternoon/evening peaks can indicate effective working hours across cities, offering insights into optimizing productivity. Thus, companies or authorities could incentivize accessibility to city centres before or after peak hours, promoting the use of public services, or even rescheduling working hours for some specific job sectors.

In summary, future cities face significant challenges, including the promotion of sustainable mobility for their residents. Urban and transport planners must implement strategies that reduce on-road mobility while maintaining efficiency. This requires optimizing mobility by adopting more sustainable systems that reduce lane occupancy and environmental impact. The results presented in this article enrich the debate on urban mobility and city size, addressing complexity and linking it to the physical capacity of urban networks to absorb traffic flows. This research contributes to more informed decision-making aimed at optimizing public space, better managing on-road traffic, and rebalancing the modal share to embrace a more sustainable and diverse transportation ecosystem. These measures can alleviate traffic congestion, improve urban mobility efficiency, enhance citizens’ well-being and create more liveable urban environments.

Finally, it is important to mention that open and public access to loop detector data, exemplified by initiatives like UTD19, along with the protocols developed to analyse these data, may contribute to a transformable shift in urban governance. This facilitates knowledge sharing and the implementation of collaborative strategies based on a more holistic approach to effectively address future challenges in urban mobility. By adopting decision-making based on real-time traffic flows, cities can provide more efficient and scalable responses to mobility concerns.

## 7. Limitations

Nonetheless, our study has some limitations. To ensure a clear understanding of the results, we must note the following limitations:

- Limited scope. The current approach does not account for various physical and human factors influencing on-road traffic flows beyond city size, such as urban morphology, population distribution and social dynamics, among others.
- Dataset selection. All the cities presented in this study are located in affluent nations where urban growth has been regulated over time and traffic management is continuously optimized by policymakers and authorities.
- Spatial-temporal data disparities. The dataset shows significant differences in the spatial and temporal data coverage across cities, which may have affected the results. The number of loop detectors ranges from 4096 in London to 29 in Essen, while the number of measurement days ranges from 105 in Hamburg to only 6 in Kassel. This can cause distortion of the patterns due to internal phase shifts in the loop detector network, affecting in a microscopic way the correlation coefficient values obtained for the city of London. Even so, the effect of the unbalanced number and distribution of detectors is mitigated by analysing macroscopic traffic flows for entire cities. While these differences may impact statistical estimations, our approach was able to minimize the potential impact of these discrepancies.

## Data Availability

All raw data can be extracted from the Harvard dataverse [51]. All datasets have been properly referenced in the article and additional data can be found in the electronic supplementary material [73].
